# *N*-Methyl Costaricine and Costaricine, Two Potent Butyrylcholinesterase Inhibitors from *Alseodaphne pendulifolia* Gamb.

**DOI:** 10.3390/ijms241310699

**Published:** 2023-06-27

**Authors:** Muhammad Hafiz Husna Hasnan, Yasodha Sivasothy, Kooi Yeong Khaw, Mohd Azlan Nafiah, Hazrina Hazni, Marc Litaudon, Wan Adriyani Wan Ruzali, Sook Yee Liew, Khalijah Awang

**Affiliations:** 1 Chemistry Division, Centre for Foundation Studies in Science, Universiti Malaya, Kuala Lumpur 50603, Malaysia; hafiz_husna@um.edu.my (M.H.H.H.); wanadriyani@gmail.com (W.A.W.R.); 2 School of Pharmacy, Monash University Malaysia, Jalan Lagoon Selatan, Bandar Sunway 47500, Malaysia; yasodha.sivasothy@monash.edu (Y.S.); khaw.kooiyeong@monash.edu (K.Y.K.); 3 Department of Chemistry, Faculty of Science and Mathematics, Universiti Pendidikan Sultan Idris, Tanjung Malim 35900, Malaysia; azlan@fsmt.upsi.edu.my; 4 Centre for Natural Products Research and Drug Discovery (CENAR), Universiti Malaya, Kuala Lumpur 50603, Malaysia; hazrinahazni@um.edu.my; 5 Institut de Chimie des Substances Naturelles, CNRS, UPR 2301, Université Paris-Saclay, 91198 Gif-sur-Yvette, France; marc.litaudon@cnrs.fr; 6 Department of Chemistry, Faculty of Science, Universiti Malaya, Kuala Lumpur 50603, Malaysia

**Keywords:** *Alseodaphne pendulifolia* Gamb., Lauraceae, alkaloid, bisbenzylisoquinoline, aporphine, oxoaporphine, acetylcholinesterase, butyrylcholinesterase

## Abstract

Studies have been conducted over the last decade to identify secondary metabolites from plants, in particular those from the class of alkaloids, for the development of new anti-Alzheimer’s disease (AD) drugs. The genus *Alseodaphne*, comprising a wide range of alkaloids, is a promising source for the discovery of new cholinesterase inhibitors, the first-line treatment for AD. With regard to this, a phytochemical investigation of the dichloromethane extract of the bark of *A. pendulifolia* Gamb. was conducted. Repeated column chromatography and preparative thin-layer chromatography led to the isolation of a new bisbenzylisoquinoline alkaloid, *N*-methyl costaricine (**1**), together with costaricine (**2**), hernagine (**3**), *N*-methyl hernagine (**4**), corydine (**5**), and oxohernagine (**6**). Their structures were elucidated by the 1D- and 2D-NMR techniques and LCMS-IT-TOF analysis. Compounds **1** and **2** were more-potent BChE inhibitors than galantamine with IC_50_ values of 3.51 ± 0.80 µM and 2.90 ± 0.56 µM, respectively. The Lineweaver–Burk plots of compounds **1** and **2** indicated they were mixed-mode inhibitors. Compounds **1** and **2** have the potential to be employed as lead compounds for the development of new drugs or medicinal supplements to treat AD.

## 1. Introduction

Alzheimer’s disease (AD) is a progressive and irreversible neurodegenerative disease that gradually causes cognitive decline and memory loss. ACh is a cholinergic neurotransmitter in the central nervous system (CNS), which relays information in the form of an electrical impulse from one neuron to another during neurotransmission. AD disrupts this communication network in the brain through the hydrolysis and inactivation of ACh by the acetylcholinesterase (AChE) and butyrylcholinesterase (BChE) enzymes, in turn terminating the signal transmission. The inhibition of the activities of AChE and BChE, which subsequently increases the ACh concentration, has been the primary step in the treatment of AD [1,2,3,4,5,6,7].

The cholinesterase inhibitors approved by the Food and Drug Administration (FDA) to treat patients with mild to moderate AD include donepezil, galantamine, and rivastigmine. Though frequently used, the capacity of these drugs to cross the blood–brain barrier (BBB) is limited. Furthermore, these drugs have also shown various dose-associated side-effects, particularly at higher doses. Thus, there is still a need for new cholinesterase inhibitors with lower toxicity and higher BBB penetration [1,8].

Alkaloids and non-alkaloids have both been explored for their potential as cholinesterase inhibitors. Among these two classes of compounds, natural and semi-synthetic alkaloids have been found to be more promising candidates owing to their complex nitrogen-containing structures. It has been reported that one of the binding sites of AChE involves the interaction with the positively charged nitrogen. Hence, alkaloids serve as a template for the development of potent and effective new cholinesterase inhibitors [9,10]. 

During the last decade, our group has been investigating the potential of the isoquinoline-type and indole-type alkaloids from the Lauraceae and Rubiaceae as cholinesterase inhibitors. Altogether, 35 alkaloids with varying degrees of AChE and BChE inhibitory activities were isolated and characterized from the following genera: *Beilschmiedia*, *Nauclea*, and *Cryptocarya* [11,12,13,14]. Among these, 2-hydroxy-9-methoxyaporphine, laurotetanine, and liriodenine from *Beilschmiedia alloiophylla* (Rusby) Kosterm and *B. kunstleri* Gamble exhibited AChE inhibitory activities that were comparable to that of huperzine A, a prescribed drug to treat AD [13], while angustidine, nauclefine, angustine, and harmane from *Nauclea officinalis* (Pierre ex Pit,) Merr. & Chun demonstrated a significantly higher BChE-inhibiting potency compared to galanthamine [14]. *Cryptocarya griffithiana* Wight yielded 2-methoxyatherosperminine, which is considered to be a potent BChE inhibitor [11]. 

*Alseodaphne pendulifolia* Gamb. is one out of eight species within the genus *Alseodaphne* that has been studied for its chemical constituents. It has been reported to yield isoquinoline- type alkaloids such as aporphine, oxoaporphine, and bisbenzylisoquinoline [15]. However, till present, there has only been two reports on the compounds which have been isolated and characterized from this species [16,17]. Hence, exploring the chemical constituents of *A. pendulifolia* and their biological activities is valuable. 

In an attempt to further identify new and potent AChE or BChE or dual-cholinesterase inhibitors with either an isoquinoline or indole backbone, our group explored the alkaloidal content of the bark of *A. pendulifolia* and assessed their enzyme inhibitory activities. 

## 2. Results and Discussion

### 2.1. Screening of Extracts for Cholinesterase Inhibitory Activities

Preliminary screening of the dichloromethane and methanol extracts of the bark of *A. pendulifolia* at a concentration of 100 μg/mL revealed that the dichloromethane extract (47.01 ± 1.81% and 95.36 ± 0.67%, respectively) was more effective in inhibiting the activities of AChE and BChE compared to the methanol (<10% and 28.41 ± 4.2%, respectively) extract. Therefore, the dichloromethane extract was further investigated with the intention of identifying the compound(s) that were responsible for giving rise to the AChE and BChE inhibitory activities. 

### 2.2. Isolation, Purification, and Structural Elucidation of Compounds

**Figure 1:**
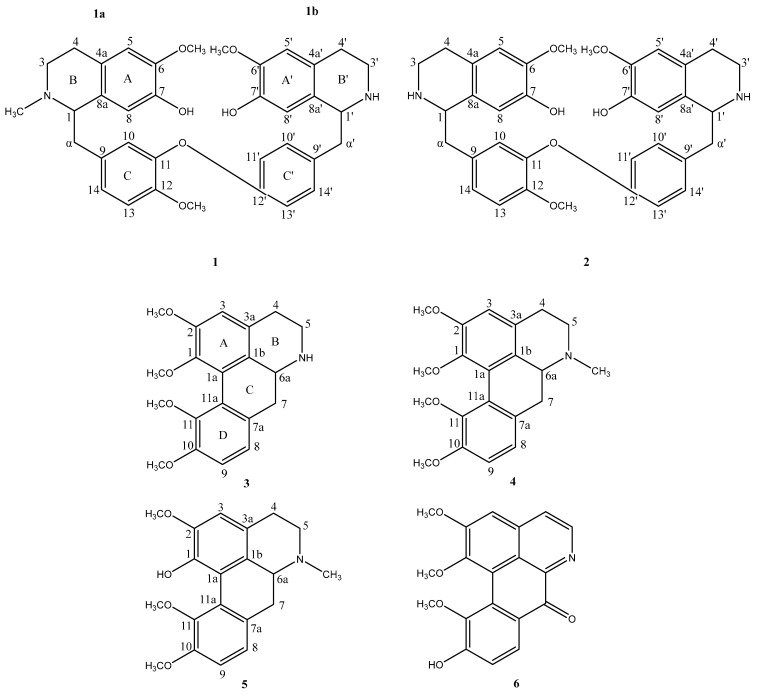
Structures of compounds **1**–**6**.

The dichloromethane extract was subjected to repeated silica gel column chromatography and preparative TLC to yield six alkaloids (**1**–**6**) among which compound **1** was identified to be a new bisbenzylisoquinoline-type alkaloid. Compounds **2**–**6** were identified as costaricine (**2**) [18], hernagine (**3**) [19,20], *N*-methyl hernagine (**4**) [20,21], corydine (**5**) [22], and oxohernagine (**6**) [21] upon comparison of their spectroscopic data with those reported in the literature (Figure 1). Compound **2**, like compound **1**, is a bisbenzylisoquinoline-type alkaloid. Compounds **3**–**5** are classified as aporphine-type alkaloids, while compound **6** is an oxoaporphine-type alkaloid.

Compound **1** (Figure 1) was isolated as an optically active yellow amorphous powder. The positive LCMS-IT-TOF analysis, which exhibited a pseudo-molecular ion [M + H]^+^ at *m*/*z* 597.2866 (Figure S1) (calcd. for C_36_H_41_N_2_O_6_ 597.2965), enabled a molecular formula of C_36_H_40_N_2_O_6_ with 18 degrees of unsaturation to be proposed. The high molecular mass and considering the fact that bisbenzylisoquinoline-type alkaloids have been characterized in the genus *Alseodaphne*, it was reasonable to postulate the possibility of compound **1** being such a compound [23,24,25].

The UV spectrum of compound **1** exhibited characteristic absorption peaks of a bisbenzylisoquinoline moiety at λ_max_ 223 and 282 nm [26,27]. The IR spectrum revealed absorption bands due to hydroxyl (ν_max_ 3536 cm^−1^), amine (ν_max_ 3306 cm^−1^), methylene (ν_max_ 2937 and 2841 cm^−1^), and aromatic (ν_max_ 1608 and 1506 cm^−1^) functional groups, consistent with those present in bisbenzylisoquinoline-type alkaloids.

The paired signals in the ^13^C NMR spectrum implied that the structure of compound **1** was not symmetrical in nature. Therefore, the structure of compound **1** was proposed to be constructed from two partial structures, **1a** and **1b**. Partial structures **1a** and **1b** each comprised three substructures, rings A-C and rings A’-C’ (Figure 1).

**Figure 2:**
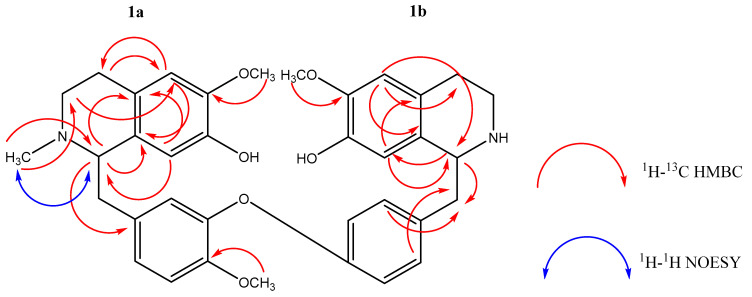
Key ^1^H-^13^C HMBC and ^1^H-^1^H NOESY correlations of compound **1**. The red arrows indicate HMBC correlations while the blue arrow indicates NOESY correlations.

Analyses of the ^1^H (Table 1, Figure S2) and ^13^C NMR spectra (Table 1, Figure S3) indicated the presence of two 1,2,4,5 tetrasubstituted aromatic rings: ring A [δ_H_ 6.46 *s* (H-5); δ_C_ 110.8 (C-5) and δ_H_ 6.30 *s* (H-8); δ_C_ 114.1 (C-8)] and ring A’ [δ_H_ 6.55 *s* (H-5′); δ_C_ 111.3 (C-5′) and δ_H_ 6.73 *s* (H-8′); δ_C_ 112.5 (C-8′)], a 1,3,4-trisubstituted aromatic ring (ring C) with a characteristic ABX spin system [δ_H_ 6.58 (*d*, *J* = 1.9 Hz, H-10; δ_C_ 121.6, C-10), δ_H_ 6.85 (*d*, *J* = 8.5 Hz, H-13; δ_C_ 112.6, C-13) and δ_H_ 6.89 (*dd*, *J* = 8.5, 1.9 Hz, H-14; δ_C_ 125.6, C-14)] and a 1,4-disubstituted aromatic ring (ring C’) with a characteristic AA’BB’ spin system [δ_H_ 7.15 (*d*, *J* = 8.5 Hz, H-10′ and H-14′; δ_C_ 130.6, C-10′ and C-14′) and δ_H_ 6.83 (*d*, *J* = 8.5 Hz, H-11′ and H-13′; δ_C_ 118.3, C-11′ and C-13′)]. The two piperidine moieties (rings B and B’) were evident from the ^13^C NMR spectrum, which revealed the following resonances: two methine carbons at δ_C_ 64.7 [C-1; δ_H_ 3.71 (*t*, *J* = 5.2 Hz, H-1)] and δ_C_ 56.8 (C-1′; δ_H_ 4.18 (*dd*, *J* = 9.4, 3.8 Hz, H-1′) and four methylene carbons at δ_C_ 47.7 (C-3), δ_C_ 40.8 (C-3′), δ_C_ 25.4 (C-4), and δ_C_ 28.6 (C-4′). The ^1^H and ^13^C NMR spectra also exhibited signals at δ_H_ 2.84–2.90 (*m*, H-*α*_a_ and H-*α*_a_’), δ_H_ 3.05–3.13 (*m*, H-*α*_b_), and δ_H_ 3.17 (*dd*, *J* = 14.0, 3.8 Hz, H-*α*_b_’), which were attributable to the pair of methylene protons bonded to C-*α* (δ_C_ 40.1) and C-*α*’ (δ_C_ 41.5) (Figure S4). The singlets at δ_H_ 3.80, δ_H_ 3.82, and δ_H_ 3.81 in the ^1^H NMR spectrum, assignable to the methyl carbon resonances at δ_C_ 55.9, δ_C_ 56.2, and δ_C_ 56.0 in the ^13^C NMR spectrum, were ascribed to the three methoxyl groups at 6-OCH_3_, 12-OCH_3_, and 6′-OCH_3_, respectively. The connectivity between substructures A-C in partial structure **1a** and substructures A’-C’ in partial structure **1b** and the position of the methoxyl groups in rings A, A’, and C were unambiguously established using the HMBC experiment (Table 1, Figure 2, Figure S5). The four quaternary aromatic carbons at δ_C_ 144.2 (C-7), δ_C_ 145.3 (C-11), δ_C_ 144.1 (C-7′), and δ_C_ 156.4 (C-12′) suggested that they were oxygenated.

Further analysis of the ^1^H and ^13^C NMR spectroscopic data of compound **1** revealed them to be comparable with those of compound **2**. There was, however, a significant difference between ring B of compound **1** upon comparison with the corresponding substructure of compound **2**. Unlike the latter, whose nitrogen atom was bonded to a hydrogen atom, the corresponding nitrogen atom in compound **1** was connected to a methyl group instead. The presence of a methyl group in the structure was apparent from the resonances at δ_H_ 2.51 (*s*, 3H, 2-*N*-CH_3_) and δ_C_ 42.5 (2-*N*-CH_3_) in the ^1^H NMR and ^13^C NMR spectra of compound **1**, respectively. The downfield shifts in the resonances of the C-1 and C-3 signals in compound **1** with respect to the corresponding atoms in compound **2** initially suggested the possibility of the methyl group being bonded to the nitrogen atom. The long-range heteronuclear correlations between 2-*N*-CH_3_ with C-1 and C-3 as inferred from the HMBC experiment in addition to the NOE interactions between 2-*N*-CH_3_ and H-1 substantiated the position of the methyl group in the molecule (Table 1, Figure 2). Based on this evidence, compound **1** was an analogue of compound **2**, hence making it a new bisbenzylisoquinoline alkaloid, trivially named *N*-methyl costaricine.

Compounds **3** and **4** have similar structures, except the latter has a methyl group bonded to the nitrogen atom. Besides, the structure of compound **4** is also very similar to compound **5**, except that the former has a methoxyl group at C-1 instead of a hydroxyl group. Meanwhile, compound **6** is an oxoaporphine, which has a fully conjugated isoquinoline skeleton. Moreover, it has a carbonyl group at C-7 instead of a methylene carbon and a methoxyl group at C-10. Compounds **3** and **6** were first isolated from *Hernandia nymphaeifolia* (Presl) Kubitzki (Hernandiaceae) [20], while compound **4** was first reported in *Parabenzoin praecox* (SIEB. *et* ZUCC.) NAKAI (Lauraceae) [28]. Compound **2**, which has a similar skeleton as compound **1** has only been identified in three species: *Nectandra salicifolia* (H.B.K.) Nees (Lauraceae) [18], *Menispermum dauricum* DC. (Menispermaceae) [29], and *Alseodaphne pendulifolia* Gamb. [16]. Compound **5** on the other hand, is a well-known aporphine alkaloid, which has been reported in many plants, particularly in the families Annonaceae [30,31], Papaveraceae [32,33,34], Ranunculaceae [35,36], Menispermaceae [37,38], and Lauraceae [39].

### 2.3. Cholinesterase Inhibitory Activities of Compounds

The initial cholinesterase inhibitory activities of compounds **1**–**5** were assayed at 100 μM (Table 2). Compounds **1** and **2** exhibited more than 70% inhibition towards both AChE and BChE. Compounds **3**–**5**, on the other hand, only exhibited more than 70% inhibition towards BChE. These compounds were further evaluated in order to determine their respective IC_50_ values. The IC_50_ values and the selectivity indices of compounds **1**–**5** and the standard drug, galantamine, are presented in Table 2.

Compounds **1** (3.51 ± 0.80 µM) and **2** (2.90 ± 0.56 µM) exhibited significantly higher BChE-inhibiting potentials compared to compounds **3** (32.92 ± 0.99 µM), **4** (49.67 ± 3.95 µM), and **5** (52.95 ± 4.11 µM). It is interesting to note that compounds **1** and **2** were two-fold more potent than galantamine (6.97 ± 0.05 µM) itself. The order of the BChE inhibitory activity of the isolated compounds in relation to galantamine was **2** > **1** > galantamine > **3** > **4** > **5**. As for the activity against AChE, compounds **1** (22.76 ± 1.25 µM) and **2** (17.50 ± 2.16 µM) were both moderate inhibitors, with the latter being the stronger inhibitor. 

A closer look at the structures of compounds **1**–**5** provided further insight as to how the cholinesterase enzyme inhibitory activities of these alkaloids might have been influenced by the chemical groups in their respective structures. Although compound **1** is almost identical in structure to compound **2**, the presence of a methyl group bonded to the nitrogen atom in ring B could have slightly suppressed its AChE and BChE inhibitory activities in comparison to compound **2** in which the methyl group was absent (Figure 1). The BChE inhibiting potential of compounds **3**–**5** though considered to be moderately active, exhibited varying degrees of potency. Although compounds **3**–**5** each possessed an aporphine nucleus with a 1,2,10,11-tetra substituted biphenyl system, it is evident that the methyl group bonded to the nitrogen atom in ring B of compounds **4** and **5** and the hydroxyl group bonded to position C-1 of ring A in compound **5** could have caused the decrease in their respective BChE inhibitory activities compared to the activity of compound **3** in which the methyl group was absent and a methoxyl group occupied position C-1 instead (Figure 1).

With regard to the selectivity of the compounds (Table 2), compounds **1** and **2** were found to be BChE selective inhibitors in contrast to galantamine, which is an AChE selective inhibitor.

### 2.4. Enzyme Kinetic Study

**Figure 3:**
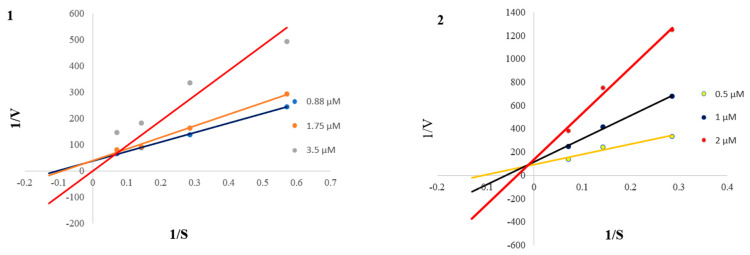
Lineweaver−Burk plots of cholinesterase inhibition activities of compounds **1** and **2**.

Kinetic studies were subsequently carried out on compounds **1** and **2**, which actively inhibited the BChE, in order to determine their mode of inhibition. As illustrated in the Lineweaver–Burk plot analyses (Figure 3), compounds **1** and **2** both displayed mixed-mode inhibition against BChE as indicated by their data lines, which either intersected in the first (for compound **1**) or second (for compound **2**) quadrants. This type of inhibitor is able to bind to the active site of the enzyme, as well as at different sites of the enzyme (allosteric site) due to the allosteric effect [40,41]. The inhibition constants, K_i_, were derived from the secondary plots for compounds **1** (0.67 µM) and **2** (0.05 µM), implying that compound **2** has a 13-fold higher affinity to BChE compared to compound **1**.

## 3. Materials and Methods

### 3.1. General Experimental Procedures

Analytical and preparative TLC was carried out on Merck 60 F254 silica gel plates (absorbent thickness: 0.25 and 0.50 mm, respectively) (Merck, Darmstadt, Germany). Column chromatography (CC) was performed using silica gel 60 (70–230 mesh, ASTM) (Merck, Germany). All solvents were of analytical-grade and were distilled prior to use. IR spectra were recorded using a Perkin-Elmer Spectrum 400 FT-IR Spectrometer. NMR spectra were acquired in CDCl_3_ with tetramethylsilane as an internal standard (Merck, Germany) using either a JOEL ECX 500 MHz NMR Spectrometer or a BRUKER Advance III 600 NMR Spectrometer (BRUKER, Billerica, MA, USA). LCMS-IT-TOF spectra were obtained using an Agilent 6530 Accurate-Mass Q-TOF LC/MS system (Agilent, Santa Clara, CA, USA). UV spectra were recorded using a Shimadzu UV-250 UV-Vis Spectrophotometer (Shimadzu, Tokyo, Janpan). A Jasco P-1020 polarimeter was used to record the optical rotation (JASCO, Hachioji, Tokyo, Japan).

### 3.2. Plant Material

*A. pendulifolia* was collected from the Sungai Tekam Reserve Forest, Jerantut, Pahang, Malaysia. The plant was identified by a botanist, Mr. Teo Leong Eng, and a voucher specimen (KL5732) has been deposited with the University of Malaya herbarium. 

### 3.3. Extraction and Isolation of Compounds ***1***–***6***

The dried powdered bark of *A. pendulifolia* (2.0 kg) was initially defatted with n-hexane (12 L, 3x) at room temperature for three days. The residual plant material was next dried and moistened with 25% ammonia solution for two hours, following which it was extracted with CH_2_Cl_2_ (12 L, 3x) and subsequently with methanol (12 L, 3x) at room temperature for three days, yielding 145 g and 30 g of extracts, respectively. Then, 38 g of the CH_2_Cl_2_ extract was chromatographed over a silica gel column (950 g, 7.5 × 68.8 cm) eluting with EA gradually enriched with CH_3_OH (0–18%, 1.0 L for each solvent mixture), followed by 1.0 L of 100% CH_3_OH to provide five major fractions. Purification of fraction 2 (5.694 g) using prep-TLC [CH_2_Cl_2_: CH_3_OH (98:2 *v*/*v*, saturated with NH_4_OH)] led to the isolation of **5** (220 mg). Prep-TLC of fraction 3 (12.076 g) with CH_2_Cl_2_:CH_3_OH (96:4 *v*/*v*, saturated with NH_4_OH) afforded **3** (950 mg), **4** (105 mg), and **6** (89 mg). Final purification to yield **2** (180 mg) and **1** (102 mg) was achieved via prep-TLC of fractions 4 (6.445 g) and 5 (6.125 g), respectively, with CH_2_Cl_2_:CH_3_OH (96:4 *v*/*v*, saturated with NH_4_OH) as the developing solvent. 

#### *N*-Methyl Costaricine (1)

Yellow amorphous powder, [*α*]_D_ −52.5° (*c* 2.0, MeOH); UV λ_max_ (MeOH) nm (log ε): 223 (4.23) and 282 (4.01); IR ν_max_ (NaCl) cm^−1^: 3536, 3306, 2937, 2841, 1608, 1506; LCMS-IT-TOF *m*/*z*: 597.2866 [M + H]^+^ (calcd. for C_36_H_41_N_2_O_6_ 597.2965). ^1^H and ^13^C NMR spectroscopic data: see Table 1.

### 3.4. In Vitro Cholinesterase Enzyme Inhibitory Activity

The cholinesterase inhibitory activities of the extracts and fompounds **1**–**6** were performed as previously described [42]. AChE from *Electrophorus electricus* and BChE from equine serum were purchased from Sigma-Aldrich Co. LLC. (St. Louis, MO, USA). An amount of 0.1 M of sodium phosphate buffer (pH 8) was added to a 96-well microplate followed by the sample and 1U of the AChE or BChE. Then, 10 mM of 5,5′-dithiobis(2-nitrobenzoic acid) (DTNB) was added followed by 14 mM of acetylthiocholine iodide or S-butyrylthiocholine chloride. The final concentration of DMSO was 1%. Galanthamine was used as the standard for validation and result comparison. The absorbance was measured using a Tecan Infinite 200 Pro Microplate Spectrometer at 412 nm for 30 min. 

### 3.5. Enzyme Kinetic Studies

The enzyme kinetic studies of compounds **1** and **2** against BChE were performed in a similar manner to the in vitro BChE inhibition assay. The enzyme inhibition kinetic was carried out in the presence of three concentrations of the inhibitors, compounds **1** and **2**, at various concentrations of the substrate, S-butyrylthiocholine chloride by constructing a Lineweaver–Burk plot (reciprocal plots of 1/V versus 1/[S]). The inhibition constant (Ki) was estimated from the secondary plot of the Lineweaver–Burk plot [14]. 

## 4. Conclusions

*N*-methyl costaricine (**1**), a new bisbenzylisoquinoline alkaloid, along with costaricine (**2**), hernagine (**3**), *N*-methyl hernagine (**4**), corydine (**5**), and oxohernagine (**6**) were isolated and characterized from the bark of *A. pendulifolia*. The AChE level decreases significantly and the ratio of BChE to AChE changes dramatically in the cortical regions with AD progression [43,44]. Hence, a BChE inhibitor could have better curative effects for AD. In this study, compounds **1** and **2** displayed potent BChE inhibitory activity. Therefore, the potency of compounds **1** and **2** in the current investigation suggested that further studies can be carried out to evaluate their amyloid-β-inhibition potential in vitro, and these alkaloids could be employed as lead compounds for the development of new drugs or medicinal supplements to treat AD.

## Figures and Tables

**Table 1:** ^1^H NMR and ^13^C NMR spectroscopic assignments of compound **1** (^1^H: 600 Mz; ^13^C: 150 MHz; *δ* in ppm; *J* in Hz) in CDCl_3_-*d*_1_.

Position	δ_H_^a^	δ_C_	COSY	HMBC
1	3.71 *t* (5.2)	64.7	H-*α*_a_, H-*α*_b_	C-3, C-4a, C-8, C-8a, C-9, C-*α*
3	*α*, 2.69–2.76 *m* ^b^ *β*, 3.05–3.13 *m* ^c^	47.7	H-3*β*, H-4*α*, H-4*β* H-3*α*, H-4*α*, H-4*β*	C-1, C-4, C-4a, C-5, C-*α* C-1, C-4, C-4a, C-8a, C-*α*
4	*α*, 2.53–2.57 *m* *β*, 2.69–2.76 *m* ^b^	25.4	H-3*α*, H-3*β*, H-4*β* H-3*α*, H-3*β*, H-4*α*	C-1, C-4a, C-5 C-1, C-3, C-4a, C-5
4a		124.9		
5	6.46 *s*	110.8		C-4, C-6, C-7, C-8, C-8a
6		145.9 ^e^		
7		144.2		
8	6.30 *s*	114.1		C-1, C-4a, C-5, C-6, C-7
8a		128.4		
*α*	*a*, 2.84–2.90 *m* ^d^ *b*, 3.05–3.13 *m* ^c^	40.1	H-*α*_b_, H-1 H-*α*_a_, H-1	C-1, C-4a, C-8a, C-9, C-10, C-14 C-1, C-4a, C-8a, C-9, C-10, C-14
9		131.9		
10	6.58 *d* (1.9)	121.6		C-12, C-14, C-*α*
11		145.3		
12		149.4		
13	6.85 *d* (8.5)	112.6	H-14	C-9, C-11
14	6.89 *dd* (8.5, 1.9)	125.6	H-13	C-10, C-12, C-*α*
2-*N*-CH_3_	2.51 *s*	42.5		C-1, C-3
6-OCH_3_	3.80 *s*	55.9		C-6
12-OCH_3_	3.82 *s*	56.2		C-12, C-13
1′	4.18 *dd* (9.4, 3.8)	56.8	H-*α*_a_’, H-*α*_b_’	C-8a’, C-α’
3′	*α*, 2.98 *m* *β*, 3.27 *m*	40.8	H-3*β*’, H-4*α*’, H-4*β*’ H-3*α*’, H-4*α*’	C-1′, C-4′, C-4a’, C-*α*’ C-1′, C-4′, C-4a’
4′	*α*, 2.69–2.76 *m* ^b^ *β*, 2.84–2.90 *m* ^d^	28.6	H-3*α*’, H-3*β*’, H-4*β*’ H-3*α*’, H-4*α*’	C-3′, C-4a’, C-5′ C-1′, C-3′, C-4a’
4a’		125.8		
5′	6.55 *s*	111.3		C-1′, C-4′, C-6′, C-7′, C-8′, C-8a’
6′		145.8 ^e^		
7′		144.1		
8′	6.73 *s*	112.5		C-1′, C-4a’, C-6′
8a’		129.5		
*α*’	*a*, 2.84–2.90 *m* ^d^ *b*, 3.17 *dd* (14.0, 3.8)	41.5	H-*α*_b_’, H-1′ H-*α*_a_’, H-1′	C-1′, C-3′, C-4a’, C-9′ C-9′, C-10′, C-14′
9′		132.4		
10′	7.15 *d* (8.5)	130.6	H-11′	C-11′, C-12′, C-13′, C-14′, C-*α*’
11′	6.83 *d* (8.5)	118.3	H-10′	C-9′, C-12′, C-13′
12′		156.4		
13′	6.83 *d* (8.5)	118.3	H-14′	C-9′, C-11′, C-12′
14′	7.15 *d* (8.5)	130.6	H-13′	C-10′, C-11′, C-12′, C-13′, C-*α*’
6′-OCH_3_	3.81 *s*	56.0		C-6′

^a^ Coupling constants (*J*) in Hz are indicated in parentheses. ^b,c,d^ Overlapping signals. ^e^ Chemical shifts are interchangeable.

**Table 2:** Cholinesterase inhibition activities of compounds **1**–**5** and galanthamine.

Compounds	Percentage Inhibition at 100 µM ^a^		IC_50_ (µM) ^a^		Selectivity Index	
	AChE	BChE	AChE	BChE	AChE ^b^	BChE ^c^
**1**	88.86 ± 1.34	96.02 ± 1.13	22.76 ± 1.25	3.51 ± 0.80	0.15	6.48
**2**	88.33 ± 0.25	96.26 ± 0.41	17.50 ± 2.16	2.90 ± 0.56	0.17	6.03
**3**	<10	88.87 ± 0.56	NT	32.92 ± 0.99	-	-
**4**	23.14 ± 8.39	77.76 ± 3.73	NT	49.67 ± 3.95	-	-
**5**	29.73 ± 1.85	77.87 ± 1.73	NT	52.95 ± 4.11	-	-
Galantamine	NT	NT	2.78 ± 0.03	6.97 ± 0.05	2.51	0.40

^a^ Data presented as mean ± SD (n = 3). ^b^ Selectivity for AChE is defined as IC_50_ (BChE)/IC_50_ (AChE). ^c^ Selectivity for BChE is defined as IC_50_ (AChE)/IC_50_ (BChE). NT = not tested.

## Data Availability

Not applicable.

## Supplementary Materials

The following supporting information can be downloaded at: https://www.mdpi.com/article/10.3390/ijms241310699/s1.
