# Supplementary material for: N-Methyl Costaricine and Costaricine, Two Potent Butyrylcholinesterase Inhibitors from Alseodaphne pendulifolia Gamb
Source: Int J Mol Sci. 2023 Jun 27;24(13):10699. doi: 10.3390/ijms241310699 (PMC10341795; doi:10.3390/ijms241310699)
Supplement: Supplementary file 1 [file ijms-24-10699-s001.zip › ijms-2458095-supplementary.pdf]

Supplementary Material

N-Methyl Costaricine and Costaricine, Two Potent Butyrylcholinesterase Inhibitors from *Alseodaphne pendulifolia* Gamb.

Muhammad Hafiz Husna Hasnan [1,†], Yasodha Sivasothy [2,†], Kooi Yeong Khaw [2], Mohd Azlan Nafiah [3], Hazrina Hazni [4], Marc Litaudon [5], Wan Adriyani Wan Ruzali [1], Sook Yee Liew [1,4,*] and Khalijah Awang [4,6,*]

[1] Chemistry Division, Centre for Foundation Studies in Science, Universiti Malaya, Kuala Lumpur 50603, Malaysia; hafiz_husna@um.edu.my (M.H.H.H.); wanadriyani@gmail.com (W.A.W.R.)

[2] School of Pharmacy, Monash University Malaysia, Jalan Lagoon Selatan, Bandar Sunway 47500, Malaysia; yasodha.sivasothy@monash.edu (Y.S.); khaw.kooiyeong@monash.edu (K.Y.K.)

[3] Department of Chemistry, Faculty of Science and Mathematics, Universiti Pendidikan Sultan Idris, Tanjung Malim 35900, Malaysia; azlan@fsmt.upsi.edu.my

[4] Centre for Natural Products Research and Drug Discovery (CENAR), Universiti Malaya, Kuala Lumpur 50603, Malaysia; hazrinahazni@um.edu.my

[5] Institut de Chimie des Substances Naturelles, CNRS, UPR 2301, Université Paris-Saclay, 91198 Gif-sur-Yvette, France; marc.litaudon@cnrs.fr

[6] Department of Chemistry, Faculty of Science, Universiti Malaya, Kuala Lumpur 50603, Malaysia

* Correspondence: joeyliew5382@um.edu.my (S.Y.L.); khalijah@um.edu.my (K.A.)

† These authors contributed equally to this work.

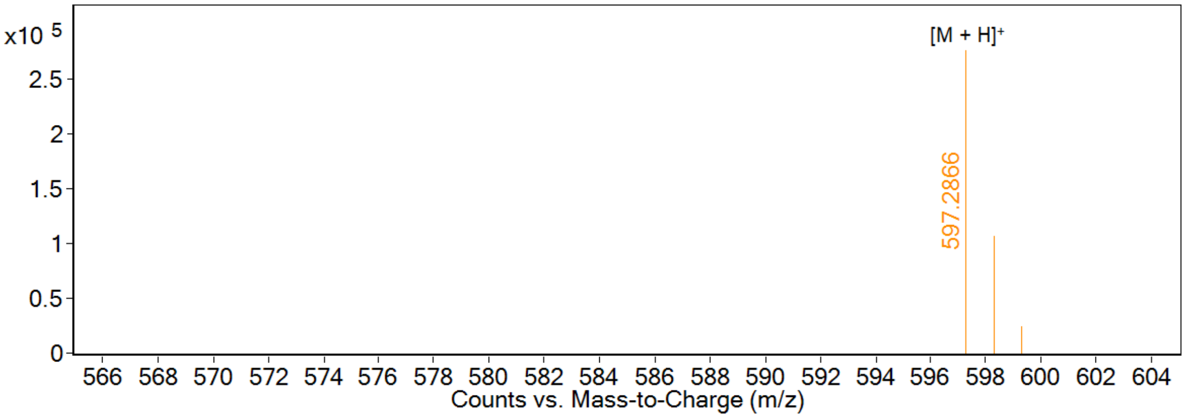

Figure S1. HRESIMS spectrum of compound **1**.

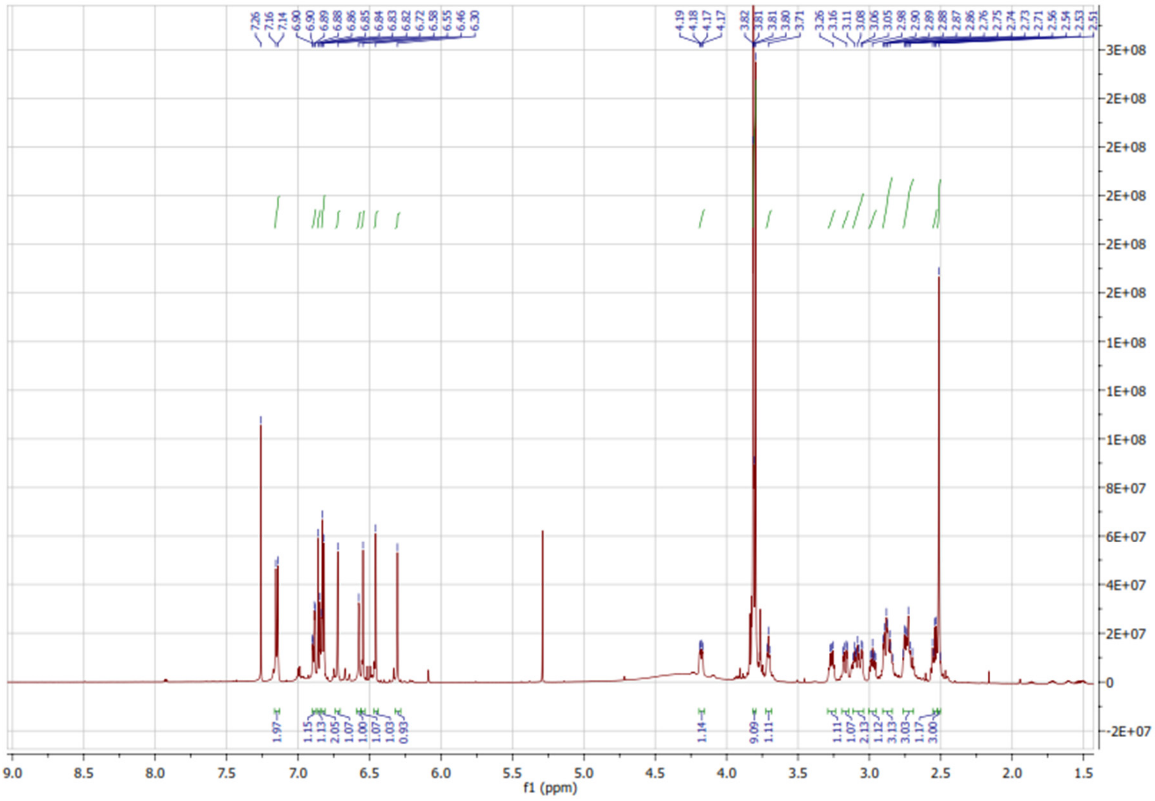

Figure S2. ^{1}H NMR spectrum of compound **1**.

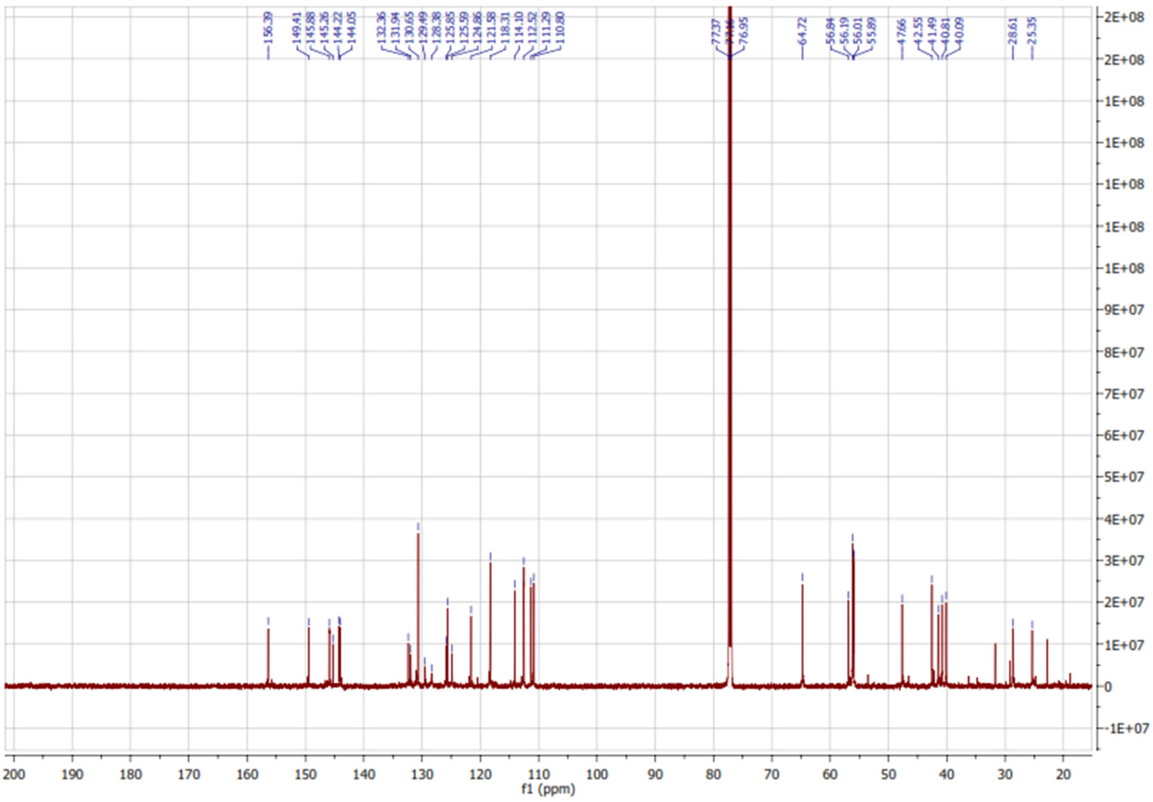

Figure S3. ^{13}C NMR spectrum of compound **1**.

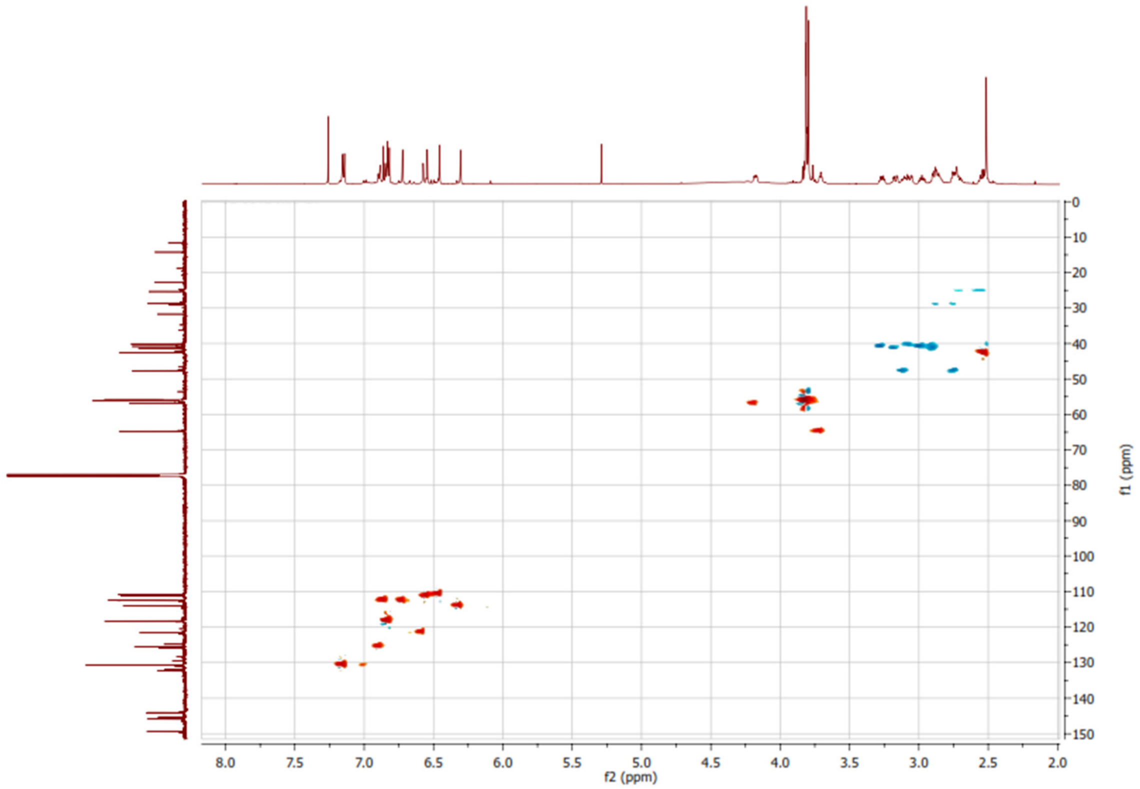

Figure S4. HSQC NMR spectrum of compound **1**.

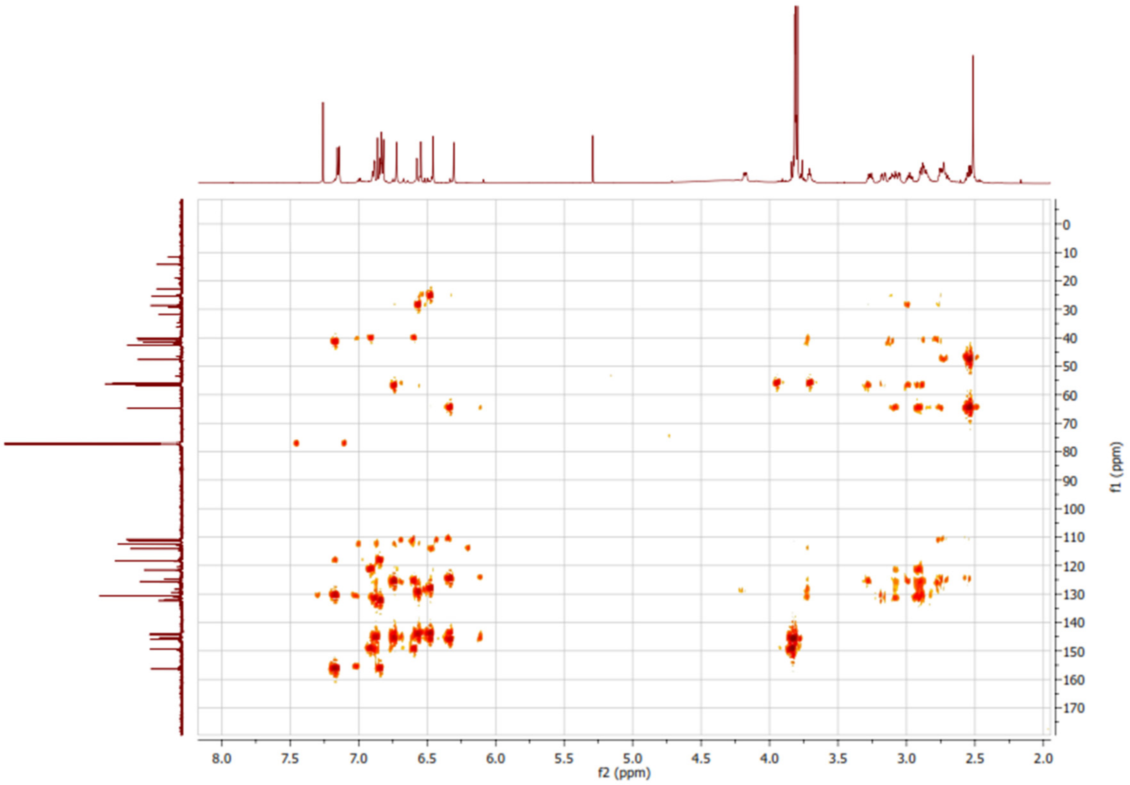

Figure S5. HMBC NMR spectrum of compound **1**.
